# Gene markers of cellular aging in human multipotent stromal cells in culture

**DOI:** 10.1186/scrt448

**Published:** 2014-04-28

**Authors:** Ian H Bellayr, Jennifer G Catalano, Samir Lababidi, Amy X Yang, Jessica L Lo Surdo, Steven R Bauer, Raj K Puri

**Affiliations:** 1 Tumor Vaccines and Biotechnology Branch, Division of Cellular and Gene Therapies, Center for Biologics and Evaluation Research, US Food and Drug Administration, Bethesda, MD, USA; 2 Office of Biostatistics and Epidemiology, Center for Biologics and Evaluation Research, US Food and Drug Administration, Rockville, MD, USA; 3 Cellular and Tissue Therapies Branch, Division of Cellular and Gene Therapies, Center for Biologics Evaluation and Research, US Food and Drug Administration, Bethesda, MD, USA

## Abstract

**Introduction:**

Human multipotent stromal cells (MSCs) isolated from bone marrow or other tissue sources have great potential to treat a wide range of injuries and disorders in the field of regenerative medicine and tissue engineering. In particular, MSCs have inherent characteristics to suppress the immune system and are being studied in clinical studies to prevent graft-versus-host disease. MSCs can be expanded *in vitro* and have potential for differentiation into multiple cell lineages. However, the impact of cell passaging on gene expression and function of the cells has not been determined.

**Methods:**

Commercially available human MSCs derived from bone marrow from six different donors, grown under identical culture conditions and harvested at cell passages 3, 5, and 7, were analyzed with gene-expression profiling by using microarray technology.

**Results:**

The phenotype of these cells did not change as reported previously; however, a statistical analysis revealed a set of 78 significant genes that were distinguishable in expression between passages 3 and 7. None of these significant genes corresponded to the markers established by the International Society for Cellular Therapy (ISCT) for MSC identification. When the significant gene lists were analyzed through pathway analysis, these genes were involved in the top-scoring networks of cellular growth and proliferation and cellular development. A meta-analysis of the literature for significant genes revealed that the MSCs seem to be undergoing differentiation into a senescent cell type when cultured extensively. Consistent with the differences in gene expression at passage 3 and 7, MSCs exhibited a significantly greater potential for cell division at passage 3 in comparison to passage 7.

**Conclusions:**

Our results identified specific gene markers that distinguish aging MSCs grown in cell culture. Confirmatory studies are needed to correlate these molecular markers with biologic attributes that may facilitate the development of assays to test the quality of MSCs before clinical use.

## Introduction

Multipotent stromal cells, also defined as mesenchymal stem cells (MSCs), undergo sustained growth *in vitro* and can give rise to cells of multiple lineages, such as adipocytes, chondrocytes, and osteoblasts [1-3]. MSC-based therapies hold potential in the field of regenerative medicine by combining elements of tissue engineering and immunosuppression to treat indications of human disorders, such as organ failure, traumatic limb injuries, genetic disorders, graft-versus-host disease, cardiovascular disease, and autoimmune disease. Hundreds of clinical trials are actively recruiting patients with specific ailments to investigate the safety and efficacy of MSCs [4,5].

MSCs can be isolated from a number of different tissues, including adipose, dermis, skeletal muscle, menstrual blood, and umbilical cord blood, but are most notably derived from bone marrow [6-12]. According to a consensus of the International Society of Cellular Therapy (ISCT), MSCs have been classified by the common characteristics of (a) adherence to plastic in standard cell-culture conditions; (b) combination of positive and negative expression of cell-surface markers (CD105+, CD73+, CD90+, CD45-, CD34-, CD14-, CD11b-, CD79α-, CD19-, and HLA-DR); and (c) *In vitro* differentiation into osteoblasts, chondrocytes, and adipocytes, as demonstrated by cell-culture staining [13]. Classification of MSCs has been further explored in the areas of additional phenotypic expression markers (CD29+, CD166+, CD133-), the benefits of immunomodulation, and precursory differentiation of cells along the ectoderm and endoderm lineage, as well as their isolation from different tissue sources [14-19].

As a heterogeneous population, MSCs have made product characterization a challenging task for investigators. The heterogeneous population of MSCs is most likely the result of contaminating cells because of the variability in isolation methods and culturing procedures, which can greatly influence their phenotype. Attempts have been made to reduce heterogeneity through separation of cells by adhesion characteristics, flow cytometry, or immunomagnetic separation [20-22]. Several studies have found that MSCs isolated from different tissue sources, including bone marrow, adipose tissue, and umbilical cord blood, have varying gene-expression profiles, which results in different trilineage cell-differentiated outcomes [23-25]. Furthermore, variation in the behavior of MSCs isolated from the same tissue sources are observed for different donors [26-30].

For some cell applications, MSC passaging and expansion in cell culture is necessary to generate sufficient numbers for transplantation. It is not clear what impact extensive cellular passaging and expansion have on the biologic activity of MSCs and in their clinical use. It has been reported that MSC populations become more homogeneous with serial passaging; however, this leads to senescent cell behavior and an impaired capacity for multipotent differentiation [31,32]. Additionally, increases in cell size and telomere shortening are commonly associated with aging cells in culture [33-36]. Other variables such as the donors’ age, body mass, gender, environment, and medical history may have a profound impact on a population of MSCs for a given therapeutic use [37,38]. These confounding variables indicate the necessity of markers that aid in rapid prediction of MSC quality and safety for a desired function. The present work aims to identify potentially predictive gene markers of human bone marrow-derived MSCs indicating cell aging through two-color gene-expression microarray technology. We hypothesize that specific gene markers exist that will distinguish aging MSCs grown in cell culture.

## Methods

### Cultivation and expansion of human bone marrow-derived MSCs

Human MSCs from six different donors were purchased from ALLCELLS and Lonza (Table 1). Because these cells were commercially available, no patient consent or approval from the FDA Research Involving Human Subject Committee was needed. Flow-cytometry analysis provided from each company revealed a marker profile of CD29+, CD44+, CD105+, CD166+, CD14-, CD34-, and CD45- for each donors’ MSCs. Cell viability was greater than 85% for all donors. MSCs were cultured in expansion medium containing αMEM (alpha minimum essential media) (Life Technologies, Grand Island, NY), 10% FBS (fetal bovine serum) (JM Bioscience, San Diego, CA), 1% L-glutamine (Life Technologies), and 1% penicillin G and streptomycin (Life Technologies) under a humidified atmosphere of 5% CO_2_ at 37°C, according to Lo Surdo *et al.*[39]. After reaching 80% confluence, cells were detached from the flask by using 0.25% trypsin/1 m*M* EDTA solution (Life Technologies) and replated at a density of 60 cells/cm^2^. The cells were expanded until the end of the seventh passage and snap frozen after passages 3, 5, and 7.

**Table 1:** Donor characteristics and cell passage

**Number**	**Donor**	**Sex**	**Age**	**Passages**	**Company**
1	PCBM1641	M	23	3, 5, 7	ALLCELLS
2	PCBM1632	F	24	3, 5, 7	ALLCELLS
3	167696	F	22	3, 5, 7	Lonza
4	110877	M	22	3, 5, 7	Lonza
5	8F3560	F	24	3, 5, 7	Lonza
6	PCBM1662	F	31	3, 5, 7	ALLCELLS

### Sample preparation: RNA isolation

Total RNA isolation was performed for the six donors at passages 3, 5, and 7 (18 different samples) by using the Qiagen RNeasy Mini Kit as per the manufacturer’s instructions. RNA quality was analyzed with the Agilent 2100 bioanalyzer if the average RNA integrity value (RIN) was 9.9 ± 0.16. The RNA concentration was measured by using the Nanodrop 1000 spectrophotometer.

### Microarray hybridization

Microarrays were produced containing 35,035 70-mer oligonucleotide probes that represented 25,100 unique genes and 37,632 transcripts, as previously described [40]. All slides were produced in our laboratory and quality tested.

**Figure 1:**
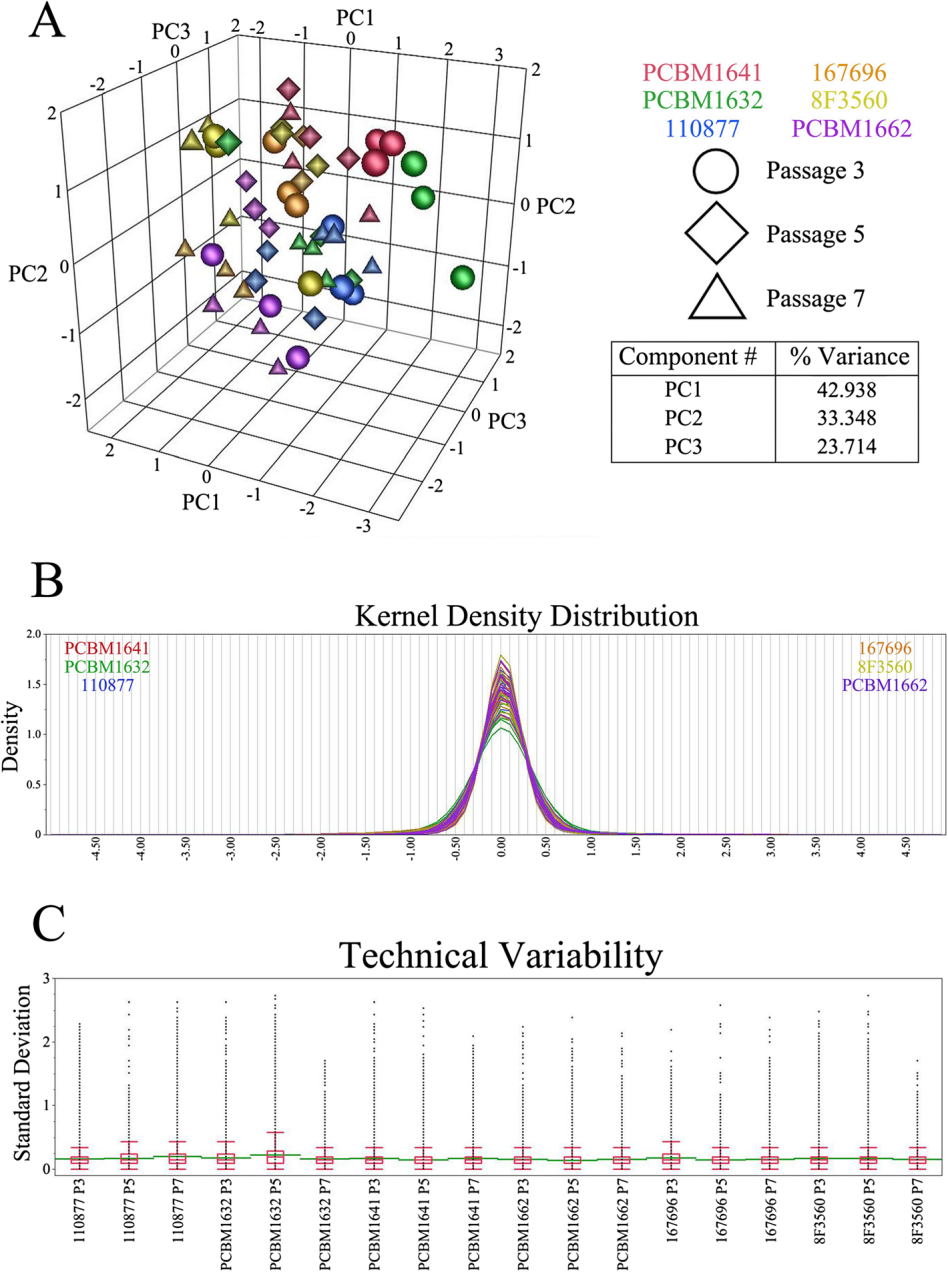
**Global microarray analysis of gene expression. (A)** Unsupervised three-dimensional representations of principal components 1, 2, and 3 for each microarray for the six donors cultured to passage 7, run in triplicate, for a total of 54 microarrays. Each symbol (circle, diamond, triangle) represents one sample on one microarray containing 35,035 probes. **(B)** Kernel density plots of normalized data for each of the donor/passage replicates. **(C)** Box-plot distributions of the technical variability per probe for each donor/passage.

Total RNA from each donor at each passage (5 μg) was reverse transcribed to the corresponding cDNA, as previously described [41]. The reference material for the arrays was composed of equal quantities of reverse-transcribed passage 3 RNA from donors 1 through 6. Sample cDNA and reference materials were labeled with Hyper5 and Cy3 reactive dye (GE Healthcare, Piscataway, NJ), respectively, in 0.1 *M* sodium bicarbonate, pH 9.1, in the dark for 1.5 hours. After purification by MinElute column, Hyper5- and Cy3-labeled cDNA were combined and mixed with the hybridization solution (Life Technologies). The combined cDNA/hybridization solution was pipetted over the entire surface of the slide and hybridized overnight (16 hours) at 42°C with the Maui hybridization system. Slides were washed for 4 minutes in both buffers 1 (1× SSC (Life Technologies) with 0.1% SDS (Sigma-Aldrich, St. Louis, MO)) and 2 (0.1× SSC), and then dried for 2 minutes during centrifugation. Samples from each donor at each passage were run in triplicate in a randomized block design to reduce possible nuisance factors (Figure 1A).

### Data acquisition and analysis

Microarray images were collected by using the 4000B GenePix Axon Scanner (Axon Instruments Inc., Union City, CA). Slides were scanned at 532 nm for Cy3 and 635 nm for Hyper5 with an image resolution of 5 μm. Spots were annotated by aligning them to the gene-array list (Gal file) that was generated according to the printing orientation, and the data were acquired by GenePix Pro. ArrayTrack software was used to normalized data by using linear and Lowess methods by using the median intensities [42]. JMP Genomics 3 software was used to perform all calculations and statistical analyses, including principal component analysis, hierarchic clustering, box plots, and the repeated measures analysis of variance (ANOVA) models. Microarray data files have been uploaded into the public repository Gene Expression Omnibus (GSE56362).

All 34,555 probes, excluding controls, were initially filtered by the technical and biologic variability and the magnitude of their expression level, as determined by the A signal (Log_2_(Cy3 × Hyper5)^1/2^). In the technical-variability filtering step, the standard deviation of the gene-expression ratio, Log_2_(Hyper5/Cy3), was calculated between technical replicates of identical donors/passages for each probe. The median technical variability per probe was calculated by taking the median of the 18 (six donors at three passages each) individual standard deviations.

Probes whose difference between passages 3 and 7 was less than the technical-variability cutoff were eliminated. To filter probes by their biologic variability, a paired *t* test was used between expression values at passage 3 and 7.

Probes were removed from the dataset if their *P* value was greater than or equal to 0.05. For the magnitude of expression filtering step, a background cutoff was calculated from the mean and standard deviation of the A signals from 2,489 negative controls and empty wells for all the microarrays.

Probes whose mean A signals at passage 3 and 7 were both less than the background cutoff (mean A signal plus 1 standard deviation) were eliminated [43]. After filtering, a repeated-measures ANOVA model using compound symmetry correlation structure and pairwise comparisons between passages was performed for each probe (911 probes total) by using *t* statistics after accounting for the chosen variance-covariance structure. The Cy3 expression value signal was added to each repeated-measure ANOVA model as a covariate to account for variability in the experimental units for each probe.

Multiplicity adjustment by using a *P* value threshold of <0.001 (with a corresponding false discovery rate of 0.023 [44]) was also applied for all pairwise comparisons between passages (911 probes × three pairwise comparisons = 2,733 *P* values). A Fisher Exact test was used by Ingenuity Pathway Analysis (IPA) software to calculate the range of *P* values for the each biofunction.

### Cell-proliferation assay

The Click-iT EdU cell-proliferation assay (Life Technologies) was used to evaluate cell division per the manufacturer’s instructions. In brief, MSCs from four different donors at passages 3 and 7 were plated on a 24-multiwell dish at a density of 5,000 cells/cm^2^. Once adhered, cells were mixed with the EdU reagent for both 6 and 18 hours. After the specified time in culture, MSCs were fixed and stained with Alexa Fluor 488 for EdU detection. The cell nuclei were visualized with Hoechst 33342 (Life Technologies) at a concentration of 5 μg/ml. Automated microscopy with a Nikon Ti-S inverted microscope with a 10× objective was performed to acquire fluorescence images at 15 randomized locations per well. All MSC donors, passages, and time points were repeated in quadruplicate, and CellProfiler version 2.0 was used to determine the percentage of EdU-positive cells. A paired *t* test was used to determine statistical differences in EdU expression of MSCs between passages 3 and 7 at both 6 and 18 hours in culture.

## Results

### Global microarray analysis of gene expression in MSCs

For gene-expression analysis, a randomized block design for the two-color gene expression microarrays with a reference was used to eliminate bias from microarrays performed at different times (Additional file 1: Figure S1). Linear and Lowess normalization rendered nearly identical normally distributed gene-expression signals (Log_2_(Sample/Reference)) for all the arrays with a mean centered near zero (Figure 1B). This aided in eliminating bias that may result at subsequent steps during the analysis by ensuring that differences were due to passage and not differences in signal range. An unsupervised principal-component analysis of the six cell lines expanded to passage 7 was performed, and the percentage variance for the first three principal components 1, 2, and 3, are 42.938, 33.348, and 23.714, respectively (Figure 1A). No single principal component captured a majority of the variance, indicating that no clear distinction exists between MSCs from different donors or passages. Instead, some minor clustering occurs among technical replicates. Furthermore, the technical variability per probe was very low, with a mean range of 0.1483 to 0.2257 for all donors/passages (Figure 1C, green line).

### Gene filtering

The objective of this study was to identify gene markers indicating MSC aging in culture across all donors. This provided the basis for the filtering process in which probes exhibiting differences between passage 3 and 7 were more useful in terms of identification markers than were differences observed between passages 3 and 5 or passages 5 and 7. To identify probes with consistent up- or downregulation with passage and therefore to minimize the number of candidates, three robust filtering steps were used: technical and biologic variability and their magnitude of expression (Additional file 1: Figure S1). The calculated difference in gene expression between passages 3 and 7 for a single probe may be indistinguishable, based on the limitations of the microarray technology; therefore it is essential to reduce type I errors by determining a technical-variability cutoff per probe. A technical-variability cutoff was calculated by using standard deviations of the technical replicates from 18 different samples.

**Figure 2:**
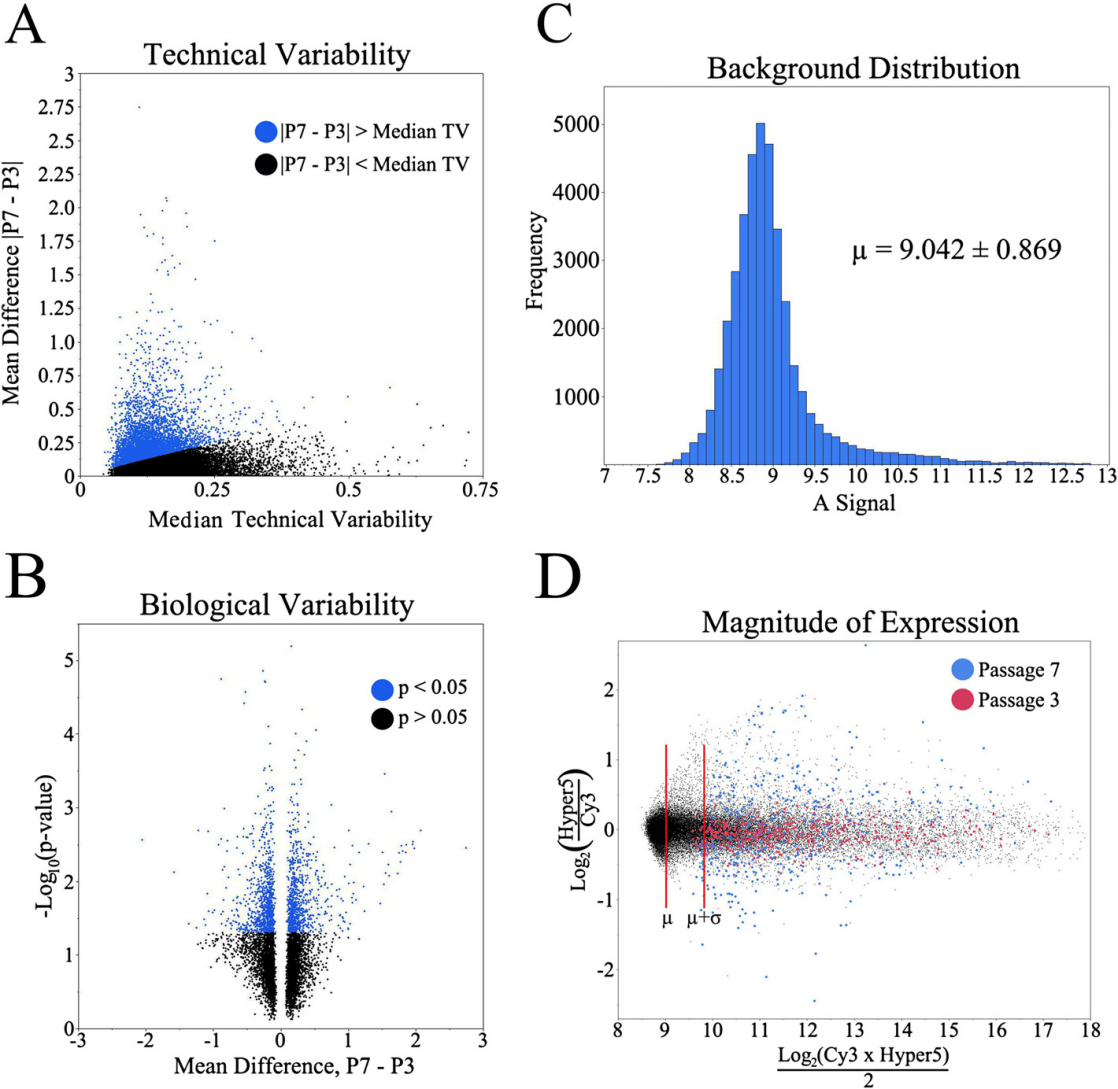
**Gene filtration. (A)** The mean difference between passage 7 and passage 3 versus the technical-variability cutoff point per probe for 34,555 probes. Blue probes (6,219) indicate that the differences between passages 7 and 3 are measurable. Blue probes were above the cutoff and used in further analysis, and black probes were below the cutoff and not used in further analysis. **(B)** Volcano plot of the mean difference between passages 7 and 3 versus –Log10 (*P* value). Blue probes (1,713) indicate that the differences between passages 7 and 3 are greater than the biologic variability. **(C)** Distribution of the A signals of the background spots with a mean and standard deviation of 9.042 ± 0.869. **(D)** MA plot of all the probes on the microarray. The blue spots represent the mean gene expression and A signal of the 911 probes at passage 7, and the red spots represent the mean gene expression and A signal at passage 3. The mean of the background (μ) and the mean of the background plus 1 standard deviation (μ + σ) are indicated by the red vertical lines.

Probes whose absolute mean difference between passages 3 and 7, not greater than this cutoff, were eliminated from the dataset, resulting in 6,219 probes (Figure 2A).

Other investigators commonly choose an arbitrary fold-change cutoff of 2 as a method to distinguish two conditions as technical replication with microarrays, because microarrays are costly [45,46]. Although this is generally a good rule of thumb, it often necessitates verification by real-time quantitative PCR to ensure the technical variability of the system using microarrays is not greater than twofold itself.

The other problem is that genes exhibiting a less than twofold change may be of biologic value, but they are often overlooked because of choosing an arbitrary cutoff. Because each sample in these experiments was repeated in triplicate, the technical variability can be calculated to eliminate genes with low fold changes, but still have detectable differences between two conditions (that is, passage 3 and 7 cells).

Biologic variability between MSCs from different donors can have a major impact on the identification of marker genes. Likewise, it is beneficial to eliminate genes whose differences between passages 3 and 7 are highly variable between donors, because the goal is to find probes that can be used as reproducible gene markers. These remaining probes were then filtered by their biologic variability by means of a paired *t* test (α = 0.05) between passages 3 and 7. This eliminated probes in which the differences in gene expression between passages 3 and 7 of individual donors were highly variable, thus resulting in 1,713 probes (Figure 2B).

To filter probes based on the magnitude of their expression, the background A signal was calculated for empty wells and negative controls, where the signal should be zero. The background signal was normally distributed, with a mean and standard deviation of 9.042 ± 0.869 (Figure 2C). Probes whose mean A signal was not greater than the background cutoff at passage 3 or 7 were eliminated from the dataset. If the mean A signal was greater than the background cutoff for only one of the passages, then the probe was considered expressed. The mean gene expression and A signal for 911 probes that passed each filtering step are visualized on an MA (log of the ratio versus log of the mean) plot for passages 3 and 7 (Figure 2D).

### Identification of gene markers as a result of cell culture

**Figure 3:**
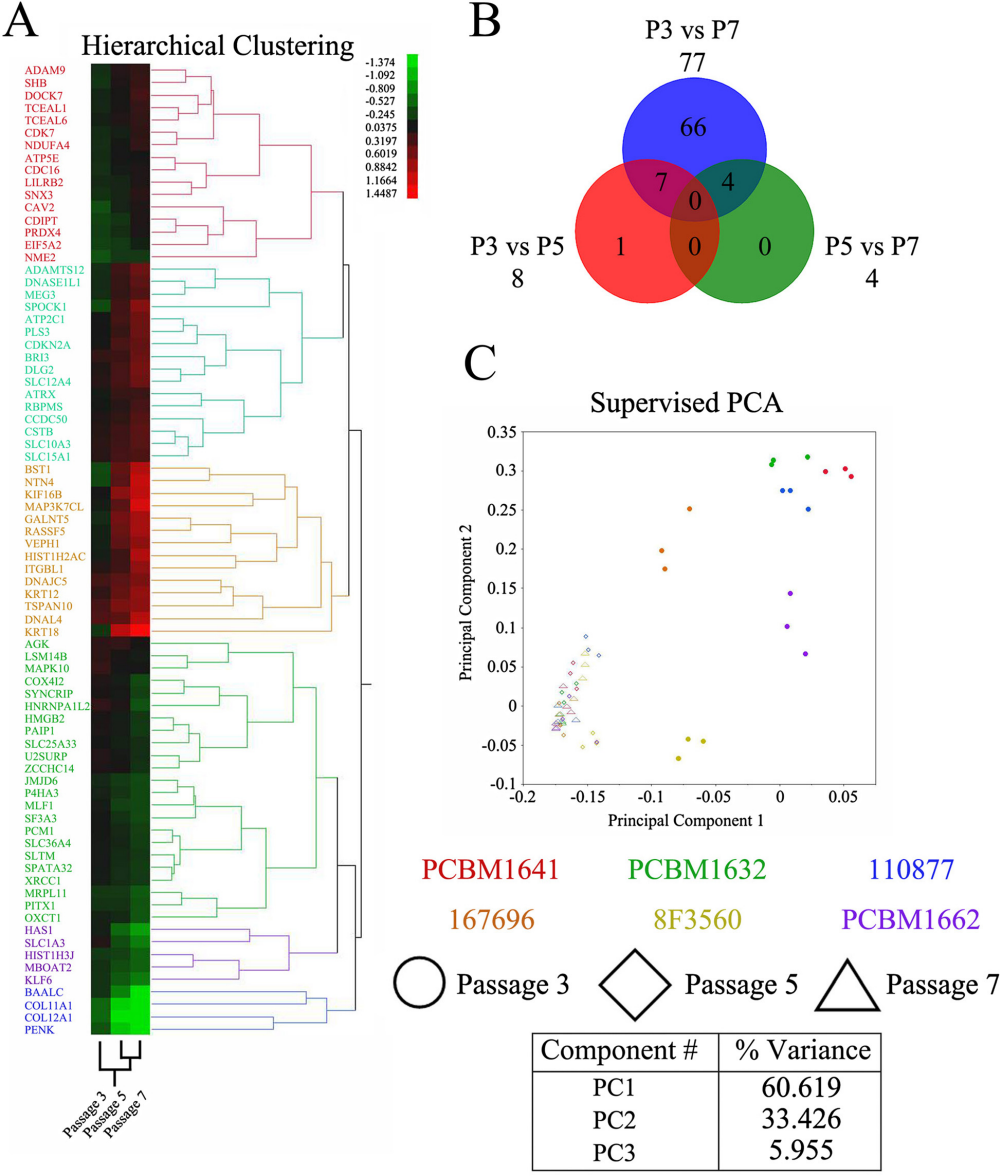
**Identification of gene markers. (A)** Hierarchic clustering heatmap of the 78 unique gene markers of passage. **(B)** Venn diagram of the 78 significantly different genes between each passage. **(C)** Supervised principal-component analysis based on the 99 significantly different probes representing the 78 unique gene markers.

The MSCs expressed the ISCT markers CD29+, CD44+, CD73+, CD90+, CD105+, and CD166+ consistently through cell passaging, with no statistically significant differences observed [47]. With a repeated-measures ANOVA with pairwise comparisons between passages and multiplicity adjustment, the data resulted in 99 statistically significant probes. In total, the 99 probes amounted to 81 genes that were significantly different between passages 3 and 7, where three genes are of an unknown classification (Table 2). Significant changes were not observed for any of the ISCT criteria markers; CD105+ (*ENG*), CD73+ (*NT5E*), CD90+ (*THY1*), CD45- (*PTPRC*), CD34-, CD14-, CD11b- (*ITGAM*), CD79α- (*IGA*), CD19- (*B4*), and HLA-DR, nor other known classification markers, CD29+ (*ITGB1*), CD166+ (*ALCAM*), and CD133- (*PROM1*). Four genes, *KRT18* (16), *ITGBL1* (2), *NTN4* (2), and *RBPMS* (2), were spotted multiple times as different sequences across the microarray and determined to be statistically significant. Of the 78 known genes that were found to be significantly different, only one of the genes was not found to be significant between passages 3 and 7, whereas only eight genes were significantly different between passages 3 and 5, and three genes were significantly different between passages 5 and 7 (Figure 3B). No genes were found to be statistically significant between all combinations of passages.

**Table 2:** **A list of statistically significant (*P*** **< 0.001) genes with indicated fold changes (Log**_**2**_**(fold change))**

**Number**	**Gene accession number**	**Gene name**	**Gene symbol**	**Location**	**Mean fold change (MFC)**			**Technical variability**	**Biologic variability** **(P7/P3)** **(P5/P3)** **(P7/P5)**
					**P7/P3 (Log**_**2**_**(MFC))**	**P5/P3 (Log**_**2**_**(MFC))**	**P7/P5 (Log**_**2**_**(MFC))**		
1	NM_003816	ADAM metallopeptidase domain 9	ADAM9	Plasma membrane	1.25 (0.321)	NS	NS	0.112	0.226
									-
									-
2	XM_005248381	ADAM 7metallopeptidase with thrombospondin type 1 motif, 12	ADAMTS12	Unknown	1.59 (0.672)	NS	NS	0.143	0.477
									-
									-
3	NM_018238	Acylglycerol kinase	AGK	Cytoplasm	−1.10 (−0.133)	NS	−1.13 (−0.170)	0.128	0.110
									-
									0.163
4	NM_001199183	ATPase, Ca^2^ transporting, type 2C, member 1	ATP2C1	Cytoplasm	1.47 (0.555)	NS	NS	0.101	0.325
									-
									-
5	NM_006886	ATP synthase, H + transporting, mitochondrial F1 complex, epsilon subunit	ATP5E	Cytoplasm	1.10 (0.142)	1.10 ([0.133)	NS	0.092	0.069
									0.084
									-
6	NM_000489	Alpha thalassemia/mental retardation syndrome X-linked	ATRX	Nucleus	1.18 (0.242)	NS	NS	0.108	0.191
									-
									-
7	NM_024812	Brain and acute leukemia, cytoplasmic	BAALC	Cytoplasm	−2.33 (−1.223)	NS	NS	0.105	0.509
									-
									-
8	NM_015379	Brain protein I3	BRI3	Unknown	1.26 (0.333)	NS	NS	0.112	0.237
									-
									-
9	NM_004334	Bone marrow stromal cell antigen 1	BST1	Plasma membrane	2.46 (1.297)	NS	NS	0.134	0.595
									-
									-
10	NM_001206748	Caveolin 2	CAV2	Plasma membrane	1.35 (0.438)	NS	NS	0.207	0.309
									-
									-
11	NM_174908	Coiled-coil domain containing 50	CCDC50	Cytoplasm	1.25 (0.322)	NS	NS	0.113	0.194
									-
									-
12	NM_003903	Cell division cycle 16 homolog (*Saccharomyces cerevisiae*)	CDC16	Nucleus	1.16 (0.218)	NS	NS	0.093	0.059
									-
									-
13	NM_006319	CDP-diacylglycerol-inositol 3-phosphatidyltransferase	CDIPT	Cytoplasm	1.27 (0.345)	NS	NS	0.173	0.270
									-
									-
14	NM_001799	Cyclin-dependent kinase 7	CDK7	Nucleus	1.21 (0.278)	NS	NS	0.107	0.143
									-
									-
15	NM_001195132	Cyclin-dependent kinase inhibitor 2A (melanoma, p16, inhibits CDK4)	CDKN2A	Nucleus	1.37 (0.452)	NS	NS	0.104	0.402
									-
									-
16	NM_001854	Collagen, type XI, alpha 1	COL11A1	Extracellular space	−2.19 (−1.132)	NS	NS	0.243	0.947
									-
									-
17	NM_004370	Collagen, type XII, alpha 1	COL12A1	Extracellular space	−3.00 (−1.583)	NS	NS	0.185	0.895
									-
									-
18	NM_032609	Cytochrome *c* oxidase subunit IV isoform 2 (lung)	COX4I2	Cytoplasm	−1.33 (−0.407)	NS	NS	0.140	0.183
									-
									-
19	NM_000100	Cystatin B (stefin B)	CSTB	Cytoplasm	1.25 (0.323)	NS	NS	0.078	0.262
									-
									-
20	NG_021375	Discs, large homolog 2 (*Drosophila*)	DLG2	Unknown	1.43 (0.521)	NS	NS	0.106	0.263
									-
									-
21	NM_025219	DNAJ (Hsp40) homolog, subfamily C, member 5	DNAJC5	Plasma membrane	1.27 (0.342)	NS	NS	0.134	0.289
									-
									-
22	NM_005740	Dynein, axonemal, light chain 4	DNAL4	Cytoplasm	1.54 (0.626)	NS	NS	0.138	0.335
									-
									-
23	NM_001009933	Deoxyribonuclease I-like 1	DNASE1L1	Cytoplasm	1.47 (0.559)	NS	NS	0.102	0.336
									-
									-
24	NM_033407	Dedicator of cytokinesis 7	DOCK7	Plasma membrane	1.29 (0.370)	NS	NS	0.116	0.249
									-
									-
25	NM_020390	Eukaryotic translation initiation factor 5A2	EIF5A2	Cytoplasm	1.17 (0.227)	NS	NS	0.142	0.115
									-
									-
26	NM_014568	UDP-*N*-acetyl-alpha-D-galactosamine:polypeptide *N*-acetylgalactosaminyltransferase 5 (GalNAc-T5)	GALNT5	Cytoplasm	2.01 (1.005)	1.63 (0.705)	NS	0.074	0.620
									0.216
									-
27	NM_001523	Hyaluronan synthase 1	HAS1	Plasma membrane	−1.84 (−0.882)	−1.42 (−0.507)	NS	0.112	0.136
									0.132
									-
28	XM_005249437	Histone cluster 1, H2ac	HIST1H2AC	Unknown	1.99 (0.992)	NS	NS	0.159	0.904
									-
									-
29	NM_003535	Histone cluster 1, H3j	HIST1H3J	Nucleus	−1.22 (−0.286)	NS	NS	0.136	0.200
									-
									-
30	NM_001130688	High-mobility group box 2	HMGB2	Nucleus	−1.18 (−0.239)	NS	NS	0.082	0.135
									-
									-
31	XM_005266269	Heterogeneous nuclear ribonucleoprotein A1-like 2	HNRNPA1L2	Unknown	−1.47 (−0.557)	NS	NS	0.084	0.489
									-
									-
32	NM_004791	Integrin, beta-like 1 (with EGF-like repeat domains)	ITGBL1 ^a^(2)	Unknown	1.56 (0.639)	NS	NS	0.120	0.504
									-
									-
33	NM_015167	Jumonji domain containing 6	JMJD6	Plasma membrane	−1.16 (−0.220)	NS	NS	0.100	0.108
									-
									-
34	NG_028043	Kinesin family member 16B	KIF16B	Cytoplasm	2.04 (1.027)	1.62 (0.697)	NS	0.094	0.547
									0.175
									-
35	NM_001300	Kruppel-like factor 6	KLF6	Nucleus	−1.43 (−0.518)	NS	NS	0.148	0.086
									-
36	NM_000223	Keratin 12	KRT12	Cytoplasm	1.51 (0.598)	NS	NS	0.234	0.377
									-
37	NM_199187	Keratin 18	KRT18 ^a^(16)	Cytoplasm	3.62 (1.813)	2.34 (1.205)	NS	0.157	0.891
									0.497
									-
38	NM_001080978	Leukocyte immunoglobulin-like receptor, subfamily B (with TM and ITIM domains), member 2	LILRB2	Plasma membrane	1.11 (0.156)	NS	NS	0.151	0.147
									-
									-
39	NM_144703	LSM14B, SCD6 homolog B (*Saccharomyces cerevisiae*)	LSM14B	Unknown	−1.12 (−0.165)	NS	NS	0.116	0.118
									-
									-
40	NM_020152	MAP3K7 C-terminal like	MAP3K7CL	Unknown	2.36 (1.238)	NS	NS	0.13	0.962
									-
									-
41	NG_013325	Mitogen-activated protein kinase 10	MAPK10	Cytoplasm	−1.15 (−0.201)	−1.15 (−0.199)	NS	0.146	0.172
									0.196
									-
42	NM_138799	Membrane bound *O*-acyltransferase domain containing 2	MBOAT2	Cytoplasm	−1.26 (−0.338)	NS	NS	0.105	0.279
									-
									-
43	NR_002766	Maternally expressed 3 (nonprotein coding)	MEG3	Unknown	1.41 (0.505)	NS	NS	0.218	0.359
									-
									-
44	NM_001130156	Myeloid leukemia factor 1	MLF1	Nucleus	−1.22 (−0.289)	−1.19 (−0.256)	NS	0.126	0.126
									0.068
									-
45	NM_170738	Mitochondrial ribosomal protein L11	MRPL11	Cytoplasm	−1.17 (−0.230)	NS	NS	0.141	0.135
									-
									-
46	NM_002489	NADH dehydrogenase (ubiquinone) 1 alpha subcomplex, 4, 9 kDa	NDUFA4	Cytoplasm	1.25 (0.327)	NS	NS	0.107	0.257
									-
									-
47	NM_001018138	Nonmetastatic cells 2, protein (NM23B) expressed in	NME2	Nucleus	NS	1.13 (0.171)	NS	0.099	-
									0.156
									-
48	NM_021229	Netrin 4	NTN4 ^a^(2)	Extracellular space	2.68 (1.401)	NS	NS	0.173	0.584
									-
									-
49	NM_000436	3-Oxoacid CoA transferase 1	OXCT1	Cytoplasm	−1.29 (−0.365)	NS	NS	0.116	0.312
									-
									-
50	NM_182904	Prolyl 4-hydroxylase, alpha polypeptide III	P4HA3	Unknown	−1.19 (−0.250)	NS	NS	0.080	0.179
									-
									-
51	NM_006451	Poly(A) binding protein interacting protein 1	PAIP1	Cytoplasm	−1.22 (−0.284)	NS	NS	0.104	0.118
									-
									-
52	NM_006197	Pericentriolar material 1	PCM1	Cytoplasm	−1.20 (−0.265)	NS	NS	0.138	0.172
									-
									-
53	NM_006211	Proenkephalin	PENK	Extracellular space	−4.15 (−2.052)	NS	NS	0.161	0.915
									-
									-
54	NM_002653	Paired-like homeodomain 1	PITX1	Nucleus	−1.17 (−0.232)	NS	−1.17 (−0.236)	0.151	0.209
									-
									0.214
55	NM_001172335	Plastin 3	PLS3	Cytoplasm	1.43 (0.515)	NS	NS	0.136	0.298
									-
									-
56	NM_006406	Peroxiredoxin 4	PRDX4	Cytoplasm	1.23 (0.301)	NS	NS	0.102	0.206
									-
									-
57	NM_182663	Ras association (RalGDS/AF-6) domain family member 5	RASSF5	Plasma membrane	1.93 (0.947)	NS	NS	0.118	0.553
									-
									-
58	NM_006867	RNA-binding protein with multiple splicing	RBPMS ^a^(2)	Unknown	1.23 (0.298)	NS	NS	0.111	0.111
									-
									-
59	NM_006802	Splicing factor 3a, subunit 3, 60 kDa	SF3A3	Nucleus	−1.26 (−0.333)	NS	NS	0.130	0.284
									-
									-
60	NM_003028	Src homology 2 domain containing adaptor protein B	SHB	Cytoplasm	1.33 (0.407)	NS	NS	0.118	0.203
									-
									-
61	NM_001142392	Solute carrier family 10 (sodium/bile acid cotransporter family), member 3	SLC10A3	Plasma membrane	1.20 (0.261)	NS	NS	0.184	0.156
									-
									-
62	NM_005072	Solute carrier family 12 (potassium/chloride transporters), member 4	SLC12A4	Plasma membrane	1.34 (0.424)	NS	NS	0.118	0.278
									-
									-
63	NM_005073	Solute carrier family 15 (oligopeptide transporter), member 1	SLC15A1	Plasma membrane	1.17 (0.228)	NS	NS	0.201	0.109
									-
									-
64	NM_001166695	Solute carrier family 1 (glial high-affinity glutamate transporter), member 3	SLC1A3	Plasma membrane	−1.72 (−0.786)	NS	NS	0.183	0.352
									-
									-
65	NM_032315	Solute carrier family 25, member 33	SLC25A33	Cytoplasm	−1.12 (−0.164)	NS	NS	0.144	0.139
									-
									-
66	NM_152313	Solute carrier family 36 (proton/amino acid symporter), member 4	SLC36A4	Unknown	−1.18 (−0.239)	NS	NS	0.124	0.156
									-
									-
67	NM_001013843	SAFB-like, transcription modulator	SLTM	Nucleus	−1.15 (−0.196)	NS	NS	0.122	0.146
									-
									-
68	NM_003795	Sorting nexin 3	SNX3	Cytoplasm	1.22 (0.293)	NS	NS	0.126	0.279
									-
									-
69	NM_152343	Spermatogenesis associated 32	SPATA32	Unknown	−1.17 (−0.229)	NS	NS	0.097	0.153
									-
									-
70	NM_004598	Sparc/osteonectin, cwcv and kazal-like domains proteoglycan (testican) 1	SPOCK1	Extracellular space	2.07 (1.052)	NS	NS	0.148	0.729
									-
									-
71	NM_001159673	Synaptotagmin binding, cytoplasmic RNA interacting protein	SYNCRIP	Nucleus	−1.24 (−0.312)	NS	−1.21 (−0.273)	0.100	0.139
									-
									0.169
72	NM_001006639	Transcription elongation factor A (SII)-like 1	TCEAL1	Nucleus	1.23 (0.297)	NS	NS	0.099	0.090
									-
									-
73	NM_001006938	Transcription elongation factor A (SII)-like 6	TCEAL6	Unknown	1.16 (0.218)	NS	NS	0.103	0.132
									-
									-
74	NM_031945	Tetraspanin 10	TSPAN10	Unknown	1.35 (0.435)	NS	NS	0.149	0.215
									-
									-
75	NM_001080415	U2 snRNP-associated SURP domain containing	U2SURP	Nucleus	−1.17 (0.225)	NS	NS	0.105	0.196
									-
									-
76	NM_001167917	Ventricular zone-expressed PH domain homolog 1 (zebrafish)	VEPH1	Nucleus	1.73 (0.793)	NS	NS	0.122	0.373
									-
									-
77	NM_006297	X-ray repair complementing defective repair in Chinese hamster cells 1	XRCC1	Nucleus	−1.14 (−0.190)	NS	NS	0.139	0.101
									-
									-
78	NM_015144	Zinc finger, CCHC domain containing 14	ZCCHC14	Unknown	−1.18 (−0.235)	NS	NS	0.152	0.156
									-
									-

NS, not significant. ^a^Repeated genes with different sequences (number of times), fold change, technical variability, and biologic variability (between each combination of passages), represented as the mean of repeated genes sequences. For “-” listed in the biologic-variability column, the value is not provided for a given passage if significance was not found between two passages.

In comparing gene expression between both vendors Lonza and ALLCELLS at passage 3, no statistically significant differences were found.

Hierarchic clustering by genes and passage revealed the relative changes in expression from passages 3 to 7 of the 78 different known genes (Figure 3A). The expression profile of genes at passage 5 clustered more closely with passage 7. Three clusters of genes exhibited upregulation from passages 3 to 7, whereas another three clusters exhibited downregulation from passages 3 to 7. The top upregulated genes at passage 7 compared with passage 3 were *KRT18, NTN4*, and *BST1*, with fold changes of 3.62, 2.68, and 2.46, respectively (Table 2). The top downregulated genes were *PENK, COL12A1*, and *BAALC*, with fold changes of −4.15, −3.00, and −2.33, respectively. The smallest fold changes detected for statistically significant genes were *ATP5E*, upregulated at 1.10, and *AGK*, downregulated at −1.10. A supervised principal-component analysis of the 78 statistically significant genes (represented by the 99 probes) between passages 3 and 7 reveals a clear separation between both passages, with a majority of the percentage variance captured in the first principal component with 60.619%, followed by 33.426% for principal component 2 and 5.955% for principal component 3 (Figure 3C). Principal components 1 and 2 for the passage 3 donor samples cluster closely with technical replicates; however, the distinct separation indicates the biologic variability between donors resulting from the identified genes. By passage 5, samples represented by both the first two principal components are intermixed with each other and samples at passage 7; thus MSCs exhibit a greater similarity with passaging.

IPA software was accessed on August 27, 2013, and the 78 significant genes were uploaded to their database, of which 74 genes were mapped. These were found to be distributed throughout all compartments of the cell, with 34.6% in the cytoplasm, 6.4% in the extracellular space, 21.8% in the nucleus, 16.7% in the plasma membrane, and 20.5% in an unknown location (Table 2). The top molecular and cellular functions and physiological system development and functions were predicted by the IPA software (Table 3). A majority of the significant genes were observed to have involvement in the categories of cellular development, cellular growth and proliferation, and tissue development.

**Table 3:** Ingenuity pathway analysis

**Molecular and cellular functions**^**a**^			
**Function**	***P *** **value**	**Number of genes**	**Genes**
Cell death and survival	1.33 × 10^−4^ – 4.52 × 10^−2^	15	↑ATP2C1, ↑ATRX, ↑CDK7, ↑CDKN2A, ↑DNAJC5, ↓HMGB2, ↓KLF6, ↑KRT18, ↓MAPK10, ↑NTN4, ↓PENK, ↑PRDX4, ↑RASSF5, ↓SLC1A3, ↑SPOCK1
Cellular development	8.80 × 10^−4^ – 4.89 × 10^−2^	21	↓AGK, ↑ATP2C1, ↑BST1, ↑CAV2, ↑CDC16, ↑CDKN2A, ↓COL11A1, ↑DOCK7, ↑EIF5A2, ↓HAS1, ↓HMGB2, ↓KLF6, ↑MEG3, ↑NME2*, ↑NTN4, ↓PCM1, ↓PENK, ↓PITX1, ↑RASSF5, ↑SLC12A4, ↓SLC1A3,
Cellular growth and proliferation	8.80 × 10^−4^ – 4.89 × 10^−2^	24	↓AGK, ↑CAV2, ↑CDC16, ↑CDK7, ↑CDKN2A, ↑DOCK7, ↑EIF5A2, ↓HAS1, ↓HMGB2, ↓KLF6, ↑LILRB2, ↓MAPK10, ↑MEG3, ↑NME2*, ↑NTN4, ↓PENK, ↑PRDX4, ↑RASSF5, ↓SF3A3, ↑SHB, ↑SLC12A4, ↓SLC1A3, ↑SPOCK1, ↓XRCC1
Cell cycle	1.48 × 10^−3^ – 4.52 × 10^−2^	12	↑ATRX, ↓AGK, ↑CDC16, ↑CDK7, ↑CDKN2A, ↓HAS1, ↓KLF6, ↑KRT18, ↓MLF1, ↓PCM1, ↑RASSF5, ↑SHB
Carbohydrate metabolism	3.85 × 10^−3^ – 2.82 × 10^−2^	5	↓AGK, ↑CDIPT, ↑GALNT5, ↓HAS1, ↓SLC1A3,
**Physiologic system development and function**			
**Function**	***P *** **value**	**Number of genes**	**Genes**
Respiratory system Development and function	1.48 × 10^−3^ – 1.91 × 10^−2^	3	↑CAV2, ↑CDKN2A, ↓PCM1
Skeletal and muscular system development and function	1.67 × 10^−3^ – 4.89 × 10^−2^	9	↑CAV2, ↑CDKN2A, ↓COL11A1, ↓COL12A1, ↓COX4I2, ↑DNAJC5, ↓HMGB2, ↓PITX1, ↓SYNCRIP
Tissue development	1.67 × 10^−3^ – 4.89 × 10^−2^	16	↑ADAM9, ↑ATP2C1, ↑CDKN2A, ↓COL11A1, ↓COL12A1, ↑CSTB, ↓HAS1, ↓HMGB2, ↓JMJD6, ↓KLF6, ↑KRT18, ↓MAPK10, ↑NME2*, ↑NTN4, ↓PITX1, ↓SLC1A3
Embryonic development	2.15 × 10^−3^ – 4.89 × 10^−2^	15	↑ATP2C1, ↑CDK7, ↑CDKN2A, ↓COL12A1, ↑CSTB , ↑DNAJC5, ↓HMGB2, ↓KLF6, ↑KRT18, ↑NME2*, ↑NTN4, ↓PITX1, ↑SHB, ↓SLC1A3, ↓XRCC1
Organ development	2.15 × 10^−3^ – 4.89 × 10^−2^	13	↑ADAM9, ↑ATP2C1, ↑CDKN2A, ↓COL12A1, ↑CSTB, ↑DNAJC5, ↓HMGB2, ↓KLF6, ↑KRT18, ↑NME2*, ↑NTN4, ↓PITX1, ↓SLC1A3

List of genes (up- or downregulated) involved in top functions. Fisher Exact Test was used by IPA to calculate the range of *P* values. ↑upregulated, ↓downregulated, *Not significant between passages 3 and 7.

### Comparison of gene-expression changes and cellular proliferation

**Figure 4:**
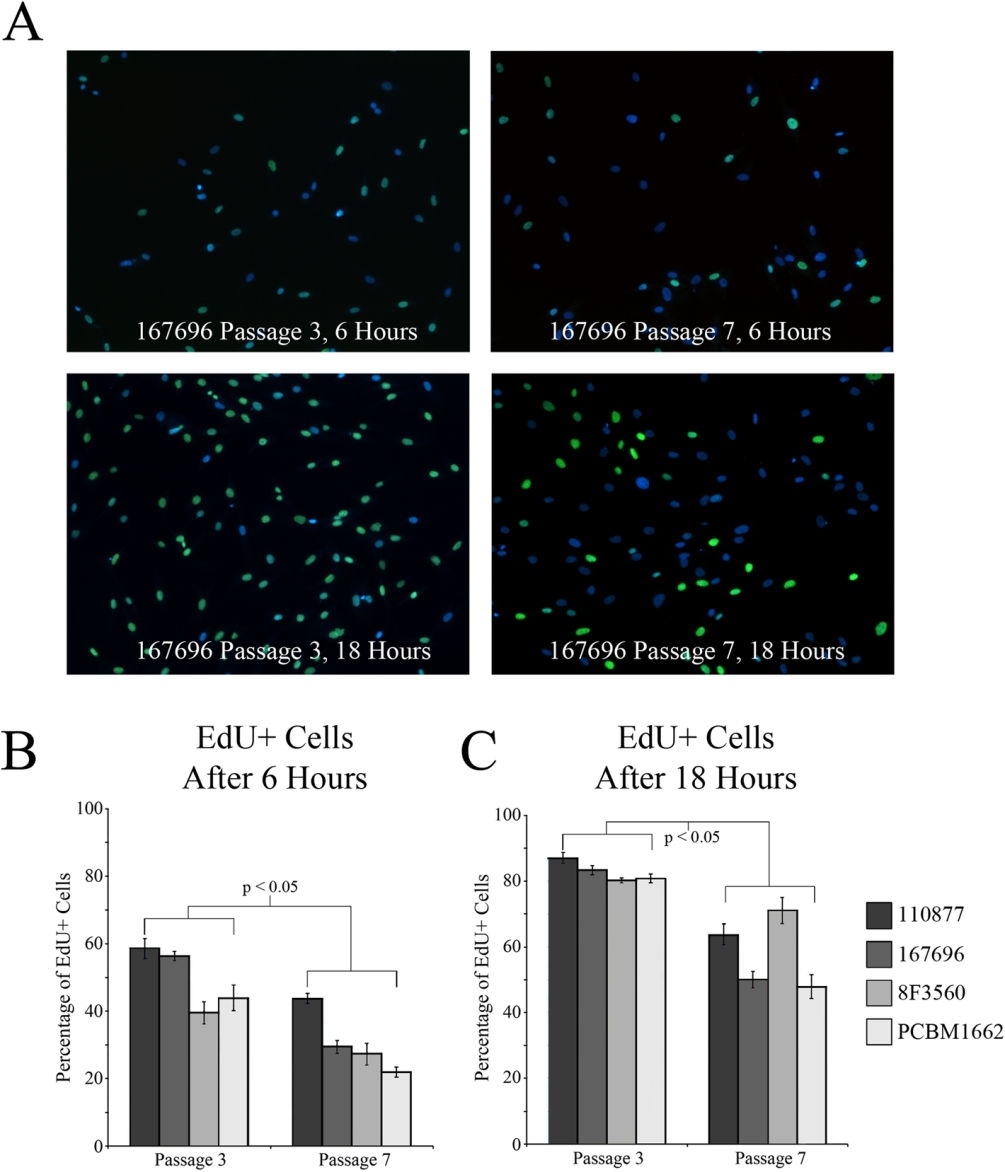
**Analysis of MSC proliferation at passages 3 and 7. (A)** The percentage of EdU-positive cells was determined for four different donors after both 6 and 18 hours at passages 3 and 7 (EdU, green;, nuclei, blue). **(B)** A paired *t* test indicated a statistically significant difference (*P* < 0.05) between passages 3 and 7 for EdU-positive expression at 6 hours. **(C)** A paired *t* test indicated a statistically significant difference between passages 3 and 7 for EdU-positive expression at 18 hours.

Because a majority of genes that were modulated as a result of cellular passage belonged to cellular growth and proliferation, we performed cell-proliferation assays on MSCs in passages 3 and 7. An EdU cell-proliferation assay was used to evaluate differences in cell proliferation between passages 3 and 7 (Figure 4A). Despite the variability in cell proliferation and percentage of cells dividing between donors at a given passage, MSCs exhibited a significantly greater potential for cell division at passage 3, as more cells incorporated EdU in comparison to passage 7, at both time points (Figure 4B,C). The fold changes in cellular proliferation between passages 3 and 7 were −1.62 and −1.42 when measured at 6 and 18 hours, respectively.

## Discussion

Gene-expression profiling by microarray technology is an excellent high-throughput method for profiling whole-genome expression of genes in any cell type and allows assessment of changes in gene expression as cells are manipulated in culture. We performed gene-expression profiling of human bone marrow-derived MSCs as they are passaged in cell culture. By highly robust statistical analysis, we discovered that 99 statistically significant probes changed expression as a result of time in cell culture. These 99 probes represented 81 unique genes that were significantly different between at least two different passages, of which 78 are known, and three have not been classified. We previously reported that these MSCs showed similar phenotypes at passages 3 and 7, despite gene-expression changes. In addition, MSCs from two different vendors exhibited uniform changes in gene expression.

For gene markers that can accurately indicate quality of MSCs in culture, markers should show a consistent pattern over time, such as an up- or downregulation, in comparison with an early time point. Genes with a temporal change in expression where expression at P5 is significantly different with P3; however, expression between P3 and P7 is not significant, and would not make an ideal gene marker. Instead, a gene marker with a significant difference between P3 and P7 would be a better predictor of MSC quality. Our filtering methods ensured that sensitivity issues (Figure 3A) and donor variation (Figure 3B) would not obscure results by eliminating genes from the dataset where expression differences between passage 3 and 7 were highly variable and not reproducible.

Combined with our multiplicity adjustment methods, we were also able to exclude genes whose statistical significance was likely due to chance. With these analytic techniques, we identified three clusters of genes that exhibited upregulation from passages 3 to 7, whereas another three clusters exhibited downregulation from passages 3 to 7.

Previous investigations have executed similar studies involving gene-expression profiling of human MSCs derived from bone marrow with long-term culture. Kulterer *et al*. reported that 838 genes were differentially expressed between P2 and P5, with 10 of those genes matching those identified in our study (Table 2) (*BST1, COL11A1, COL12A1, GALNT5, HAS1, KRT18, MEG3, PCM1, PENK*, and *SHB*) [48]. Likewise, in another study by Tanabe *et al*., two genes reported matched those found in our study (*KRT18* and *PRDX4*) [49]. A number of reasons exist for the discrepancies from a biologic standpoint, including culture conditions, media used, and the source of MSCs; however, poor experimental design and statistical analysis are the primary sources for misleading results. Ren *et al*. [50] examined the effects of cell aging on gene expression by categorizing samples by the degree of senescence in contrast to passage. This study used a highly robust statistical analysis, which identified 15 statistically significant genes (*ADAMTS12, BAALC, BST1, CAV2, CDKN2A, COL11A1, COL12A1, DNASE1L1, EIF5A2, GALNT5, KRT18, MEG3, NTN4, SLC1A3,* and *VEPH1*) that were identical to our gene list with the same up-/downregulation compared with early passages. The magnitude of fold change was also typically greater than the magnitude of fold change reported in our own results, and this may be due to Ren *et al*. carrying out cell cultures to a higher passage number. This suggests that minute differences in gene expression detected between closely related passages increases with more-distant passages or cellular aging.

Approximately 37% of the 78 gene markers imported into IPA were observed to have some function related to cellular growth and proliferation and cellular development. Two of the 78 different marker genes belong to the keratin family, KRT12 and KRT18, in which significant gene-expression changes were observed in multiple publications [48-50]. Keratins are fibrous structural proteins that play a major functional role in the integrity and mechanical stability of epithelial tissues [51]. Typically, keratins are observed in pairs such as KRT18 and KRT8, which are co-expressed in simple epithelium. As indicated in IPA (Table 3), keratins are strongly connected to the functions of cell cycle and cell death and survival, which can profoundly affect cellular development. Increases in keratin expression are particularly observed with the progression of cell differentiation in keratinocytes or in organs such as the pancreas or liver [52-56]. Sixteen different sequences representing the *KRT18* gene on these microarray chips were upregulated at passage 7 compared with passage 3. *KRT18* expression has also been suggested to play a role in the immunosuppressive potential of graft-versus-host disease (GVHD). *KRT18* expression, as measured by serum fragments, was found to be elevated in lower gastrointestinal tract and liver GVHD [57,58]. Immunosuppressive therapy for GVHD resulted in decreases of *KRT18* serum fragments.

Like *KRT18*, *NTN4*, a laminin-related secreted molecule protein exhibited more than a twofold upregulation in gene expression from passages 3 to 7. Studies suggests that *NTN4* expression is tied to decreases in cell proliferation, as has been observed in corneal and pancreatic epithelium, as well as in a human breast cancer cell line (MCF7) [59-61]. However, conflicting arguments have been made about the function of *NTN4*, as it has been observed to promote cell proliferation in various tumor cell types [62-64]. Similarly, *BST1*, bone marrow stromal cell antigen 1, was observed to have a 2.46-fold change from passages 3 to 7. It is a stromal cell line-derived glycosylphosphatidylinositol-anchored molecule that has been identified to facilitate pre-B-cell maturation based on its enhanced expression in rheumatoid arthritis-derived bone marrow stromal cell lines and activation by Pax5 [65-67]. An increase in BST1 expression has been observed in several studies with aging in culture, in which elevated expression levels have been tied to nonneurogenesis-promoting astrocytes [48,68,69].

The gene that exhibited the greatest change in expression from passages 3 to 7 was *PENK*, which was downregulated 4.15 fold. *PENK* is an endogenous opioid polypeptide hormone found at high levels in the brain and endocrine tissues [70]. They typically function as neurotransmitters to modulate pain, cellular growth, organogenesis, and immunity, because they are also widely observed in nonneuronal tissues [71]. *PENK* expression, known in early neuronal development, was observed to be upregulated in neuron-like differentiated bone marrow-derived MSCs compared with undifferentiated MSCs [72]. Likewise, *SYNCRIP*, which was downregulated from passages 3 to 7 by 1.24 fold, has been involved in neuronal synaptic transmission and was also exhibited to be upregulated in neurogenic differentiated MSCs. *PENK* expression has also been linked to osteoblastic development, in which decreases in *PENK* paralleled decreases in osteogenic differentiation, as measured alkaline phosphatase activity [73,74]. A later study has indicated that may not be true for *in vivo* experiments as *PENK*-deficient mice did not exhibit a difference in bone remodeling compared with wild-type littermates unless PENK expression was deleted from Phex-deficient Hyp mice [75].

Another gene typically involved with the developing central and peripheral nervous systems expressed to enhance growth is *KLF6*[76]. *KLF6* is known as a zinc-finger DNA-binding transcription factor regulating gene expression and was downregulated from passages 3 to 7 by −1.43. Two different studies examining *KLF6* in mice found that their expression affects neuronal morphogenesis by promoting axon outgrowth [77,78]. Furthermore, *KLF6* expression was observed to regulate proliferation and differentiation positively as KLF6−/− ES cells demonstrated significant defects after differentiation into embryoid bodies.

Like *PENK*, *BAALC* exhibited a 2.33-fold downregulation from passages 3 to 7. *BAALC* is an overexpressed gene usually found in a subset of patients with acute myeloid leukemia within neuroectoderm-derived and mesoderm tissues [79,80]. Its expression has also been observed in early hematopoietic progenitor cells, in which its subsequent loss is associated with cell differentiation [81]. Because the expression of *BAALC* was dramatically reduced with increasing passage, this suggests the MSCs lose their multipotent potential as they aged in culture. Two genes associated with fibril elongation, *COL11A1* and *COL12A1*, were also observed to be downregulated with increasing passage more than twofold. Elevated levels of expression are observed for both genes in response to different cell-proliferative assays. In hyalocytes, ascorbic acid was observed to increase their proliferation in combination with increasing *COL11A1* expression, whereas in a different study, chondrocytes exhibited high-density micromass growth in conjunction with *COL11A1*[82,83]. Similarly, *COL12A1* was upregulated with the growth of glioblastoma multiforme compared with normal brain, and when the proliferative potential of SKOV3 cells was reduced by knocking down the expression of *RUNX1*, *COL12A1* expression also decreased [84,85]. Other studies have also indicated that an elevated level of expression leads to osteoblast differentiation and maturation, whereas their suppression can terminate this process [86-88]. In our study, the decrease in both collagens with passaging suggests a reduced capacity for osteogenic differentiation or limited MSC multipotency.

The gene markers presented in this study allude to MSC senescence and unwanted differentiation that can often go unnoticed with passage. Multiple studies have demonstrated that MSCs exhibit a reduction in cellular proliferation with culture, which is consistent with our current work when these identical donors were evaluated [35,39,89]. Our previous studies examining these same donors exhibited increases in cell size with length in culture by flow cytometry and automated cell counting [39,47].

Furthermore, our prior work investigating the multipotency of these MSCs illustrated a decreased percentage of colony-forming units and a reduced potential for adipogenic differentiation, as observed by the percentage of Nile red-positive cells [39,47]. Rather than using bioassays to determine MSC quality during passaging and cell expansion, future work may establish quality through the expression of a set of gene or protein markers. Based on these results, MSCs performance is most likely best at earlier passages of culturing. The conglomerate of these gene markers exhibiting altered gene expression at passage 7 suggests that they are undergoing terminal differentiation that is comparable with reaching cellular senescence. This can negatively affect potential therapeutic applications in which the transplantation of aged cells can send detrimental signals leading to erroneous regeneration or prevent immunosuppression. Extreme caution should be considered when using the ISCT markers for MSC characterization, as they are commonly used as quality markers when generating criteria for lot release with respect to MSC identity and purity [90]. Despite the use of ISCT markers to establish purity of an MSC population, heterogeneity and diminished performance persist with continuous culturing, whereas ISCT marker expression remains consistent [47]. Our prior and current results indicate significant changes in MSC performance through biologic assays and gene expression; thus the identified gene markers may be useful in producing quality markers of cellular aging over successive rounds of passaging.

Additional work is necessary to determine whether the expression of identified gene markers is consistently modulated when cellular expansion is scaled up by using bioreactors or performed with different substrates and media components. Discovering genes expressed in MSCs that correlate with a functional outcome will provide a basis for a set of quality markers. These quality markers can then be used to assess cellular products desired for a specific application. These are major considerations in establishing quality, function, and safety of MSCs for therapeutic purposes.

## Conclusions

An increasing interest exists in determining markers that can be used to distinguish between different donors and culture conditions of MSCs. As MSCs are aged in culture, they begin to exhibit gene-expression changes related to cell growth and proliferation, cell survival, and cellular development as observed through reduced cellular proliferation at a higher passage. Reproducible gene-expression changes were consistently detected among different MSC donors. Results obtained from these studies may provide insights into MSC differentiation and function with passaging in culture.

## Abbreviations

αMEM: alpha-Minimum essential media; ANOVA: analysis of variance; FBS: fetal bovine serum; GVHD: graft-versus-host disease; IPA: ingenuity pathway analysis; ISCT: International Society for Cellular Therapy; MSC: multipotent stromal cell.

## Competing interests

The authors declare that they have no competing interests.

## Authors’ contributions

IHB was involved in the experimental conception and design, data acquisition, data analysis, interpretation, and drafting/revising the manuscript for important intellectual content. JGC was involved in the experimental conception and design, data analysis, interpretation, and drafting/revising the manuscript for important intellectual content. SL performed data analysis and drafting/revising of the manuscript for important intellectual content. AXY was involved in microarray platform manufacturing, data acquisition, and drafting/revising the manuscript for important intellectual content. JLL was involved in sample preparation, data acquisition and drafting/revising the manuscript for important intellectual content. SRB was involved in conception, design, data acquisition, and drafting/revising the manuscript for important intellectual content. RKP was involved in the conception and design, interpretation, and drafting/revising the manuscript for important intellectual content. All authors read and approved the final version of this manuscript, and agree to be accountable for all aspects of the work in ensuring that questions related to the accuracy or integrity of any part of the work are appropriately investigated and resolved.

## Supplementary Material

Additional file 1: Figure S1 Outline of the major topics and interrelated steps used to analyze microarray data. (1) Experimental design, (2) normalization, (3) gene filter, (4) statistical analysis, and (5) pathway analysis. Click here for file
