# Supplementary figures and images for: Gene markers of cellular aging in human multipotent stromal cells in culture
Source: Stem Cell Res Ther. 2014 Apr 28;5(2):59. doi: 10.1186/scrt448 (PMC4055144; doi:10.1186/scrt448)

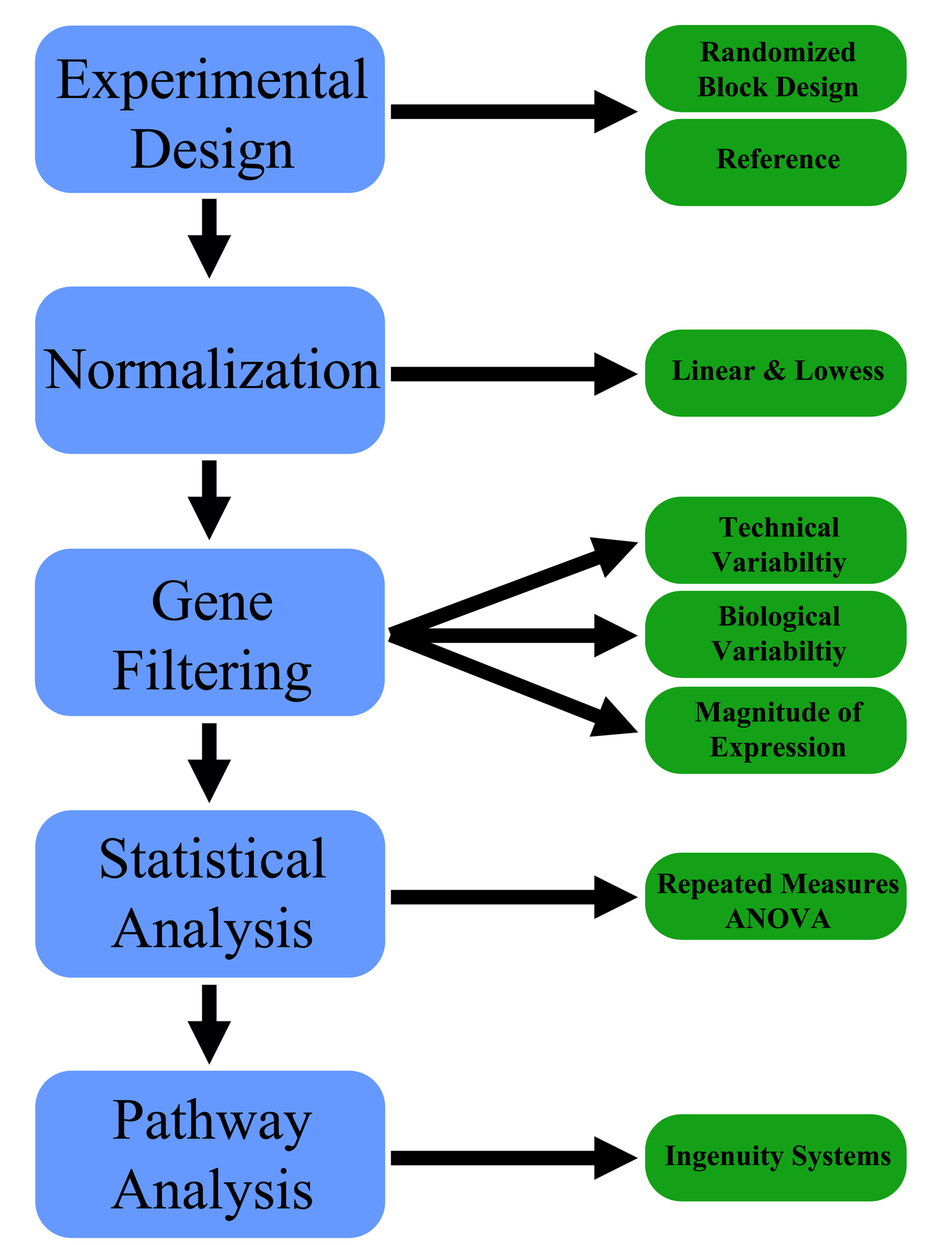

Supplement: Additional file 1: Figure S1 — Outline of the major topics and interrelated steps used to analyze microarray data. (1) Experimental design, (2) normalization, (3) gene filter, (4) statistical analysis, and (5) pathway analysis. [file scrt448-S1.tiff]
